# Food safety in a globalized world

**DOI:** 10.2471/BLT.15.154831

**Published:** 2015-04-01

**Authors:** Keiji Fukuda

**Affiliations:** a World Health Organization, avenue Appia 20, 1211 Geneva 27, Switzerland.

Access to sufficient safe food is a basic requirement for human health. Ensuring food safety and security in a highly globalized world presents increasingly difficult, and often under-appreciated challenges, for governments, commercial organizations and individuals alike.^1^^,^^2^

The risks of unsafe food are substantial, but can be difficult to quantify. Diarrhoeal diseases – both foodborne and waterborne – kill an estimated two million people annually, including many children in developing countries. Food contaminants, such as harmful parasites, bacteria, viruses, prions, chemical or radioactive substances, cause more than 200 diseases – ranging from infectious diseases to cancers.^3^

In parallel with the increasing size of the world population, consumer demand for a wider variety of foods is growing, entailing a longer and more complex food-chain. In this context, for the World Health Day, on 7 April 2015, the World Health Organization (WHO) has chosen to focus on food safety. Today, food ingredients often come from multiple countries, with each item having travelled thousands of kilometres from a field, farm or factory. Contamination at one end of the food-chain can affect populations on the other side of the world. Given the interaction of multiple actors separated by vast distances and potentially delayed impacts, multisectoral and international cooperation is essential. Food safety needs strengthening in many countries – but no country can do this alone.

World Health Day is one of a series of actions that WHO is taking to raise awareness about the food safety agenda and to galvanize action. WHO, in collaboration with the United Nations Food and Agriculture Organization (FAO), has had a central international role in developing guidelines to strengthen and harmonize food systems, in particular through the jointly managed Codex Alimentarius Commission. Codex standards have become the de facto international standards for food safety. WHO and FAO also manage the International Food Safety Authorities Network (INFOSAN), which provides timely information during food safety emergencies^4^ and assists countries in building strong systems to prevent such incidents. WHO has also established the Global Foodborne Infections Network^5^ to promote integrated, laboratory-based surveillance and foster multisectoral collaboration.

In 2010, the 63rd World Health Assembly adopted a resolution to advance food safety.^6^ As a result, a strategic plan was developed which requires WHO to: (i) provide the evidence base for measures to decrease foodborne health risks along the entire food-chain; (ii) improve international and national cross-sectoral collaboration, including communication and advocacy; and (iii) provide leadership and assist in the development and strengthening of risk-based, integrated national systems for food safety.^7^ In November 2014, the second International Conference on Nutrition^8^ reaffirmed the right for everyone to have access to safe, sufficient and nutritious food; the need to strengthen food production and distribution systems and the importance of fair trade practices. Recently, WHO has also provided guidance on food safety for food producers, transporters and consumers.^9^

There is a need to refocus attention and to re-energize commitments on food safety – especially coordinated and cooperative actions and communications across borders. Better data and methods are needed to estimate the health impact of foodborne diseases and to guide response and prevention actions. This year, WHO will release the first comprehensive estimates of the global burden of death and illness caused by foodborne diseases.^10^ More investment is needed in national food safety systems, reflecting the importance of food safety as a public health priority. Governments have several key roles to play. In addition to setting policies, they are critical for establishing and implementing the national food safety systems within which food producers and suppliers must operate. Consumers can stay informed, for example, through self-education and by reading labels on packaging.

In the 21st century, collaboration is vital to achieving safe food-chains that cross national borders. This is why WHO works closely with FAO, the World Organization for Animal Health (OIE) and other international organizations to ensure food is safe to eat. This year’s World Health Day is an opportunity to strengthen food safety across all borders and stakeholders.
